# Supplementary material for: Stacking Model‐Based Classifiers for Dealing With Multiple Sets of Noisy Labels
Source: Biom J. 2025 Mar 12;67(2):e70042. doi: 10.1002/bimj.70042 (PMC11898607; doi:10.1002/bimj.70042)
Supplement: Supplementary file 1 — Supporting Information [file BIMJ-67-e70042-s001.zip › density-based-ensemble-model-main/competitors_real_data/netlab-master/netlab-master/help/gpfwd.htm]

Netlab Reference Manual gpfwd


# gpfwd

## Purpose

Forward propagation through Gaussian Process.

## Synopsis

```
y = gpfwd(net, x)
[y, sigsq] = gpfwd(net, x)
[y, sigsq] = gpfwd(net, x, cninv)
```

## Description

`y = gpfwd(net, x)` takes a Gaussian Process data structure `net`
together
with a matrix `x` of input vectors, and forward propagates the inputs
through the model to generate a matrix `y` of output
vectors. Each row of `x` corresponds to one input vector and each
row of `y` corresponds to one output vector. This assumes that the
training data (both inputs and targets) has been stored in `net` by
a call to `gpinit`; these are needed to compute the training
data covariance matrix.

`[y, sigsq] = gpfwd(net, x)` also generates a column vector `sigsq` of
conditional variances (or squared error bars) where each value corresponds to a pattern.

`[y, sigsq] = gpfwd(net, x, cninv)` uses the pre-computed inverse covariance
matrix `cninv` in the forward propagation. This increases efficiency if
several calls to `gpfwd` are made.

## Example

The following code creates a Gaussian Process, trains it, and then plots the
predictions on a test set with one standard deviation error bars:

```
net = gp(1, 'sqexp');
net = gpinit(net, x, t);
net = netopt(net, options, x, t, 'scg');
[pred, sigsq] = gpfwd(net, xtest);
plot(xtest, pred, '-k');
hold on
plot(xtest, pred+sqrt(sigsq), '-b', xtest, pred-sqrt(sigsq), '-b');
```

## See Also

`gp`, `demgp`, `gpinit`

---

**Pages:**
Index


---

Copyright (c) Ian T Nabney (1996-9)
